# Magnitude and Mechanism of Siderophore-Mediated Competition at Low Iron Solubility in the *Pseudomonas aeruginosa* Pyochelin System

**DOI:** 10.3389/fmicb.2017.01964

**Published:** 2017-10-10

**Authors:** Konstanze T. Schiessl, Elisabeth M.-L. Janssen, Stephan M. Kraemer, Kristopher McNeill, Martin Ackermann

**Affiliations:** ^1^Department of Environmental Systems Science, Institute of Biogeochemistry and Pollutant Dynamics, Swiss Federal Institute of Technology, Zurich, Switzerland; ^2^Department of Environmental Microbiology, Swiss Federal Institute of Aquatic Science and Technology, Dübendorf, Switzerland; ^3^Department of Environmental Chemistry, Swiss Federal Institute of Aquatic Science and Technology, Dübendorf, Switzerland; ^4^Department of Environmental Geosciences, University of Vienna, Vienna, Austria

**Keywords:** siderophore, competition, iron uptake, microbial interactions, pyochelin, *Pseudomonas aeruginosa*

## Abstract

A central question in microbial ecology is whether microbial interactions are predominantly cooperative or competitive. The secretion of siderophores, microbial iron chelators, is a model system for cooperative interactions. However, siderophores have also been shown to mediate competition by sequestering available iron and making it unavailable to competitors. The details of how siderophores mediate competition are not well understood, especially considering the complex distribution of iron phases in the environment. One pertinent question is whether sequestering iron through siderophores can indeed be effective in natural conditions; many natural environments are characterized by large pools of precipitated iron, and it is conceivable that any soluble iron that is sequestered by siderophores is replenished by the dissolution of these precipitated iron sources. Our goal here was to address this issue, and investigate the magnitude and mechanism of siderophore-mediated competition in the presence of precipitated iron. We combined experimental work with thermodynamic modeling, using *Pseudomonas aeruginosa* as a model system and ferrihydrite precipitates as the iron source with low solubility. Our experiments show that competitive growth inhibition by the siderophore pyochelin is indeed efficient, and that inhibition of a competitor can even have a stronger growth-promoting effect than solubilization of precipitated iron. Based on the results of our thermodynamic models we conclude that the observed inhibition of a competitor is effective because sequestered iron is only very slowly replenished by the dissolution of precipitated iron. Our research highlights the importance of competitive benefits mediated by siderophores, and underlines that the dynamics of siderophore production and uptake in environmental communities could be a signature of competitive, not just cooperative, dynamics.

## Introduction

Bacteria rely on iron for survival and growth, apart from rare exceptions. At the same time, bacteria face low iron availability in most environments, as under aerobic, pH-neutral conditions, the solubility of the predominant iron species, ferric iron, is only around 10^-17^ M (Andrews et al., 2003). Even though the absolute iron concentration may be several magnitudes higher, iron above this solubility limit precipitates and is not available for direct uptake by bacteria. To increase iron uptake, many bacteria secrete iron-chelating molecules called siderophores (Hider and Kong, 2010). The primary role of siderophores is thought to be increasing iron availability through solubilization of precipitated environmental iron sources (Andrews et al., 2003).

Siderophore secretion has been used as an example of cooperative behavior (Velicer, 2003; West et al., 2006), since secreted siderophores are available to surrounding cells that express the cognate receptor but do not contribute to costly siderophore production (so-called “cheaters”; West and Buckling, 2003; Griffin et al., 2004; Ross Gillespie et al., 2007; Brockhurst et al., 2008). Based on the use of siderophores as a model system for cooperation in laboratory studies, the distribution of siderophore production and receptor genes in the environment has been interpreted mainly as a result of cooperative interactions (Cordero et al., 2012; Andersen et al., 2015).

However, siderophores also play a role in competition. The competitive role relates to the advantage that siderophore-sequestered iron becomes unavailable to cells without the uptake receptor (Wandersman and Delepelaire, 2004). Numerous papers have been published describing the growth inhibitory effect that secreted siderophores inflict (Kloepper et al., 1980; Vachée et al., 1997; Gram et al., 1999; Matthijs et al., 2007; Weller, 2007; Simões et al., 2008; Khare and Tavazoie, 2015). When these competitive effects of siderophores are included in eco-evolutionary models, they strongly influence the predicted evolutionary stability of siderophore production as well as optimal siderophore production levels (Niehus et al., 2017). Consequently, in a given environment the dynamics of siderophore production are not fully captured when siderophore production is solely studied as a cooperative trait, when in fact siderophores successfully mediate competition.

Despite a wealth of publications reporting the detection of competitive effects of siderophores, there is a lack of understanding of how and to which degree siderophores mediate growth inhibition in the presence of mineral phases that limit iron solubility to very low levels. This is one of the most common environments bacteria experience since in environmental systems, iron precipitation is ubiquitous and occurs in any aerobic, pH neutral environment where the iron concentration exceeds the solubility limit of 10^-17^ M (Andrews et al., 2003). In addition, precipitation of iron is also common in laboratory settings, where iron salts are often used in growth media (e.g., ferrous or ferric chloride or sulfate, largely precipitating in aerobic, pH neutral conditions). Note that this precipitated iron presents a source of iron. Solubilization of iron from the precipitated phase can counteract the competitive scavenging of dissolved iron by siderophores. This replenishing effect from the precipitated phase is facilitated by the fact that siderophores not only change the speciation of soluble iron but also influence the solubility of precipitated iron phases. On the one hand, the concentration of freely available soluble iron is reduced when siderophores sequester this iron. On the other hand, any iron bound to a siderophore can be replenished by dissolution of the precipitated iron pool, reestablishing the soluble iron up to the solubility limit (Kraemer, 2004). In previous work on siderophore-mediated competition, binding of iron by siderophores has usually been approximated solely by the siderophore binding affinity, without taking into account the coupled equilibria of iron dissolution (Buyer and Sikora, 1990; Fgaier and Eberl, 2010, 2011; Niehus et al., 2017). This discrepancy will affect estimates of the overall influence of siderophores on iron availability and the competitive advantage of its producers.

The primary function assigned to siderophores is the solubilization of iron precipitates in saturated systems with the goal to increase the concentration of bioavailable iron (Andrews et al., 2003). Therefore, siderophores can have two roles, solubilizing precipitated iron aggregates and scavenging dissolved iron. These two effects are linked to each other by kinetically controlled dissolution reactions and the rates of the dissolution reactions have important implications: If the dissolution reaction is fast, siderophores will increase the total dissolved iron concentration, but the concentration of non-siderophore bound iron remains constant because it is replenished by the dissolution reaction to equilibrium levels. Hence, the iron availability to competing strains would not be affected, and consequently, their growth would not be affected by secreted siderophores. Alternatively, if siderophores primarily bind dissolved iron and iron dissolution is slow or completely inhibited, then siderophores would bind almost all dissolved iron and deplete the non-siderophore bound available iron pool. Hence, their production could suppress the growth of competitors by reducing their access to iron, but not increase the total dissolved iron concentration. Here, we compare the two benefits of siderophore production being (a) increasing dissolved iron from precipitated phases to make it available to the siderophore producer, and (b) decreasing availability of iron to competitors by sequestration of dissolved iron. To investigate the competitive effect of siderophores in a quantitative way, we developed an experimental system with genetically engineered *Pseudomonas aeruginosa* strains that only differ in the ability to access iron chelated by the siderophore pyochelin. We complemented this empirical work with a thermodynamic model of iron dissolution that we tailored to our experimental system.

We found that in the presence of precipitated iron a low concentration of pyochelin siderophore is sufficient to inhibit growth of a competitor strain. In contrast, the effect of increasing growth by solubilizing precipitated iron was negligible. Based on thermodynamic modeling, we propose a mechanism for competitive growth mediated by dissolution kinetics: Siderophores can deplete dissolved iron temporarily but sufficiently to slow down uptake of freely dissolved iron before dissolution from the precipitated iron replenishes the dissolved iron pool sufficiently. Our data demonstrates that siderophore excretion can be an important and efficient competitive strategy at low iron solubility. This effect can under some conditions possibly exceed the importance of solubilization of precipitated iron sources. Since the nature of interactions between different microorganisms in the environment strongly influences the dynamics of microbial communities (Coyte et al., 2015; Oliveira et al., 2015; Niehus et al., 2017), understanding the competitive effects of siderophores has critical implications for our comprehension of environmental systems.

## Materials and Methods

### Strains

We used a ‘secretor’ strain (pyochelin production and uptake), a ‘recipient’ strain (no siderophore production) and a ‘non-recipient’ strain (neither siderophore production nor uptake) based on *Pseudomonas aeruginosa* PAO1 (ATCC 15692). More specifically, the secretor was knocked out in a gene necessary for production of the siderophore pyoverdine (PAO1 Δ*pvdD*) and was obtained from Pierre Cornelis’ laboratory (Ghysels et al., 2004). In this strain, one of the genes important for pyoverdine production, the gene encoding the pyoverdine synthetase PvdD, was removed. Thus, the secretor can only produce one type of siderophore, pyochelin. The recipient was a Δ*pvdD*Δ*pchEF* double-knockout in siderophore production, also from (Ghysels et al., 2004). In this strain, both the gene encoding the pyoverdine synthetase PvdD and also the genes encoding the pyochelin synthetases PchE and PchF have been removed. For the non-recipient, we used transduction to delete the *fptA* gene necessary for uptake of pyochelin. The donor strain of the *fptA*-deletion was obtained from the Washington mutant library (strain PW8162). The transduction was performed with phage E79tv2. The phage was obtained from Urs Jenal’s lab (Biozentrum Basel, Switzerland) and lysates were prepared according to the standard protocol (Malone et al., 2010). The transduction procedure also followed the published protocol (Malone et al., 2010), however, we optimized UV radiation times to the machine used (30 s for a PFU of around 5 × 10^8^/ml). Transductants were selected on 25 μg/ml Tetracycline, and tested via PCR.

To distinguish the strains in competition, they were tagged with constitutively expressed fluorophores. The GFP and mCherry marker were integrated using the Tn7 system (Lambertsen et al., 2004). All strains were tagged with both types of fluorescent proteins to control for the effect of the fluorescent protein expressed. The expression was under control of the constitutive promoter P_A1/04/03_, a derivate of the lactose promoter.

### Media and Growth Conditions

For all growth studies, we used succinate minimal medium (7.58 mM (NH_4_)_2_SO_4_, 0.81 mM MgSO_4_^∗^7H_2_O, 33.87 mM succinic acid, 25 mM HEPES). The pH was adjusted to 7.2, and after autoclaving, 10 mM K_2_HPO_4_ was added from a sterile stock solution. Directly before incubation, the media was filtered with a 0.1-μm filter to reduce the amount of precipitated background iron (Cellulose Nitrate Membranes, 0.1 μm, 47 mm diameter, Whatman). FeCl_3_ (anhydrous, 97% reagent grade) was added as iron source. 1000x stock solutions were made in 0.1 M HCl for FeCl_3_, filter sterilized, and kept at 4°C. All chemicals were obtained from Sigma Aldrich. Pyochelin standard was obtained from EMC microcollections (Tübingen, Germany), dissolved in methanol (HPLC reagent grade) and stored at -20°C. Working stocks were made with sterile nanopure water.

Strains were streaked from a frozen stock on standard Luria Broth agar (LB Lennox Sigma). Single colonies were picked and incubated for 20 h in succinate minimal medium containing 2 μM iron citrate at 37°C in an orbital shaker (220 rpm). Dilutions of these cultures were used for both monoculture and competition experiments. For all experiments, starting cell density was adjusted based on optical density (O.D.) measurements of the overnight cultures to a starting O.D. of 0.0001 at a wavelength of 600 nm.

### Monoculture Studies

For monocultures, strains were incubated in a 96-well Falcon plate and optical densities were measured every 10 min at 600 nm in a Synergy Biotek plate reader heated to 37°C with continuous shaking. Nine biological replicates were measured per strain. Maximum growth rate and lag time were extracted using a Matlab script written by Daan Kiviet, ETH Zurich (see Supplementary Information). For statistical analyses, a three-way ANOVA was conducted (see Supplementary Information). For the statistical analysis of data in **Figure 3**, we conducted a two-way ANOVA for the growth of the recipient (see Supplementary Information) using SPSS (IBM SPSS Statistics, version 23).

### Competition Studies

For competition studies, strains were incubated in 24- or 96-well Falcon plates in eight biological replicates. The initial frequency was set to 1:1 based on O.D. measurements and the initial total O.D. was set to 0.0001 at a wavelength of 600 nm. The initial as well as the final ratio of mCherry-to-GFP-tagged strains were measured via flow cytometry. In brief, cells were fixed with 2% formaldehyde, diluted 1:2000, and measured with a Gallios 3-laser flow cytometer (Beckman Coulter, United States). 100,000 events were recorded and cell clusters were computed with an automated clustering algorithm, flow Peaks (Ge and Sealfon, 2012). The clusters were assigned to GFP- and mCherry-tagged cells based on the fluorescence ratios.

### Calculation of Relative Fitness

Relative fitness was calculated as in (Ross Gillespie et al., 2007), based on the initial and final frequencies of the strains. Statistical tests were performed with R Version 3.1.2 (R Core Team, 2014).

### Pyochelin Isolation and HPLC Analysis

To isolate pyochelin, a slightly modified protocol from (Cox and Graham, 1979) was used. In brief, cells were removed by centrifugation (30 min, 5000 rpm, 4°C) followed by filtration with a 0.2 μm filter. The supernatant was acidified with HCl to pH 1.8-2. Three extractions with 1/3 volume of ethyl acetate were performed. The ethyl acetate was evaporated with nitrogen, and pyochelin was extracted from the remaining powder by addition of HPLC-grade methanol, rigorous vortexing, and centrifugation of other precipitated substances (5 min, 5000 rpm, room temperature). The supernatant was taken off and directly analyzed on a HPLC (see Supplementary Information and Figures S3–S7).

### Time Course Studies

Competition outcome and pyochelin concentration were measured at the same time with one type of strain-color combination (non-recipient:GFP, secretor:mCherry) in three biological replicates. Pyochelin was isolated as explained above. The ratios of the strains were estimated by flow cytometry of fixed cells as explained above. At low cell concentrations, fewer events were recorded with the flow cytometer, with a minimum of 5000 events. Also, at some time points the background counts of non-fluorescent cells were high (up to 50% of counts), and the fractions were corrected for this (i.e., we determined the fraction of secretor:mCherry and non-recipient:GFP among the *fluorescent* cells).

### Thermodynamic Speciation Calculations

Thermodynamic speciation calculations were performed using the PHREEQC 3 code (Parkhurst and Appelo, 2013) and the Phreeqc thermodynamic database. Stability coefficients and deprotonation constants for pyochelin were taken from (Brandel et al., 2012) and were corrected for zero ionic strength using the Davies equation. All calculations assumed a solution composition of the succinate minimal medium at a constant pH 7.2. Total concentrations of iron and of pyochelin were varied for each analysis. Precipitation of ferrihydrite in equilibrium with the solution was allowed using a solubility coefficient of 10^3.55^ (Schindler et al., 1963).

## Results

Our experimental system consisted of three *P. aeruginosa* strains with modifications to their pyochelin uptake and production systems (**Table 1**), called ‘secretor,’ ‘recipient,’ and ‘non-recipient.’ Pyochelin is the sole siderophore produced by these strains. We chose to work with pyochelin because its uptake depends on one specific receptor that can be deleted. Secretor and recipient strains were previously described in (Dumas et al., 2013). The ‘secretor’ strain can both secrete and uptake pyochelin, the ‘recipient’ is deficient in pyochelin production but not uptake, and the ‘non-recipient’, is deficient in both production and uptake of pyochelin due to a knockout in the pyochelin receptor FptA. Pyochelin is a relatively weak siderophore, so we expect any inhibitory effects observed in competition to be stronger when examined in context of siderophores with a higher iron binding affinity, such as pyoverdine. Finally, we constructed the ‘non-recipient,’ which is neither able to produce pyochelin nor to take it up due to a knockout in the pyochelin receptor FptA. As iron source, we used ferric chloride. At both concentrations used, 1 μM and 30 μM FeCl_3_, almost 99% of the iron precipitated due to the low solubility of amorphous iron(III)hydroxides at pH 7.2, as predicted by thermodynamic modeling (Supplementary Table S1).

**Table 1:** Overview of genetically engineered *Pseudomonas aeruginosa* PAO1 strains used to investigate the competitive effect of pyochelin.

Strain	Pyochelin production	Pyochelin uptake	Relevant knockouts
Secretor (S)	Yes	Yes	Δ*pvdD*
Recipient (R)	No	Yes	Δ*pvdDΔpchEF*
Non-recipient (NR)	No	No	Δ*pvdDΔpchEF ΔfptA*

### In a Kinetically Controlled System, Pyochelin Can Temporarily Induce Growth Inhibition

**FIGURE 1:**
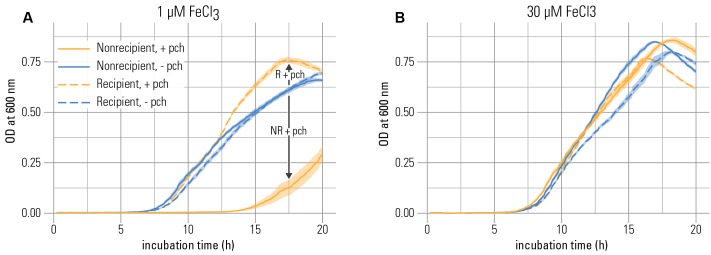
Pyochelin inhibits growth of a strain without pyochelin receptor at iron limitation. **(A)** At low iron concentration (1 μM), growth of the non-recipient strain NR (without pyochelin receptor) is inhibited upon addition of 6 μM pyochelin (+ pch; compare the blue solid line to the orange solid line). Growth of a recipient strain R with a functional pyochelin receptor is not inhibited upon addition of pyochelin (compare blue dashed line to orange dashed line). **(B)** At higher iron concentrations (30 μM), the inhibitory effect of pyochelin addition on the non-recipient is strongly reduced (compare the blue solid line to the orange solid line). Data represents mean optical density (O.D.) measurements of nine replicates; the standard error of the mean is indicated as shaded areas around the lines. The growth parameters extracted from these growth curves are depicted in Supplementary Figure S1.

We first tested whether the non-recipient was inhibited through the addition of pyochelin in the presence of precipitated iron. In line with the hypothesis that siderophore production could impose growth inhibition in strains that lack the cognate receptor, the non-recipient was affected by the presence of the siderophore (6 μM) in three key growth parameters: Its maximal growth rate and yield were reduced, and its lag phase was prolonged compared to a control without addition of pyochelin (**Figure 1A** and Supplementary Figure S1). These effects were linked to the pyochelin receptor knockout, since the recipient was not inhibited under the same conditions. Also, the inhibition was only observed for the non-recipient at low total iron concentrations (1 μM FeCl_3_; **Figure 1A** vs. **Figure 1B** and Supplementary Figure S1).

**FIGURE 2:**
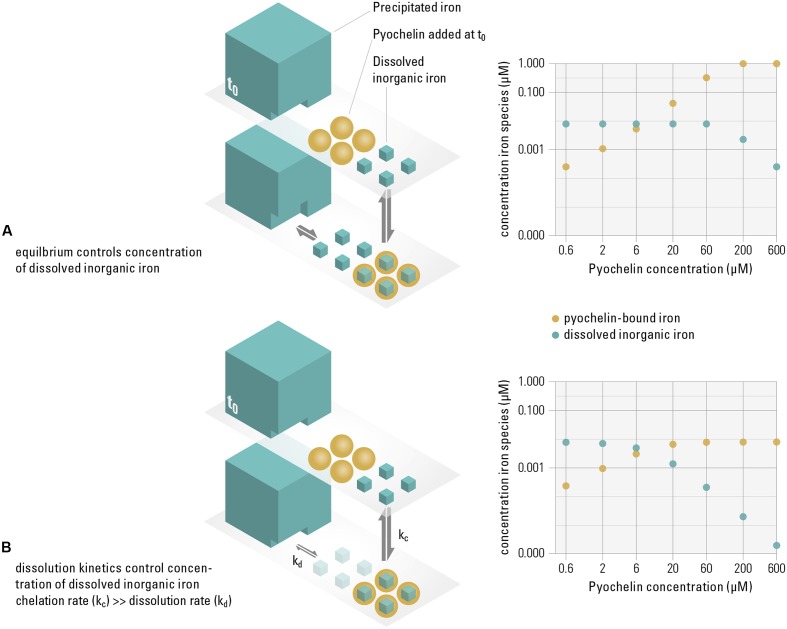
Pyochelin can only sequester dissolved iron sufficiently if the system is not in equilibrium, but controlled by slow dissolution kinetics. In our experimental system, similar to other aerobic, pH neutral environments, 99% of the iron precipitates (i.e., a saturated system), while only around 1% is present as dissolved inorganic iron, which we assume to be available to the non-recipient (t_0_). We consider the following two extreme scenarios. *Equilibrium controlled system* **(A)**: When pyochelin is added and the system is in equilibrium, the concentration of dissolved inorganic iron is buffered by the dissolution of precipitated phases. The thermodynamical model shows that even though the concentration of pyochelin-bound iron increases (yellow data points), the dissolved inorganic iron concentration remains constant at 0.08 μM (turquoise data points) up to very high pyochelin concentrations. *Kinetically controlled system* **(B)**: When iron sequestered by pyochelin is not replenished due to slow dissolution kinetics of the precipitated iron, the concentration of dissolved iron can be significantly reduced. However, if slow dissolution can partially replenish dissolved iron concentrations on the timescales of the experiments, the available iron concentrations to the non-recipient are expected to be in between these extreme scenarios illustrated. Thermodynamic modeling was used to calculate concentrations of iron species for increasing pyochelin concentrations for both scenarios (data plots on the right).

While previous reports have already linked siderophore production to growth inhibition (Simões et al., 2008; Khare and Tavazoie, 2015), our results further propose a mechanism for how siderophores successfully reduce the concentration of available iron in the presence of precipitated iron, i.e., in a saturated system. How efficiently the concentration of soluble iron is diminished by siderophores hinges on whether the system is in thermodynamic equilibrium (where sequestering efficiency depends on iron solubility and the siderophore binding affinity) or controlled by kinetics (where sequestering depends on rates, e.g., of binding or dissolving iron). By modeling the distribution of iron species in equilibrium, we can quantify if, based on iron solubility and siderophore binding affinity, pyochelin sufficiently sequesters the dissolved iron available to the non-recipient [e.g., aquo complexes such as Fe^3+^, Fe(OH)^2+^, or dissolved Fe(OH)_3_^0^]. If sufficient sequestration is not the case, the observed inhibition can only be explained if the system is controlled by kinetics. We based the calculations of the thermodynamic model on the concentration of pyochelin present in our growth experiments (from **Figure 1**). We find that in an equilibrated system, pyochelin would by far not be able to deplete the concentration of dissolved iron. Even at a ten-fold higher pyochelin concentration (60 μM) than present in the experiment, our model predicts that the dissolved fraction of iron would remain constant at 7.9 nM, i.e., at the solubility limit of ferrihydrite (**Figure 2A**). The lack of complete sequestration is caused by the establishment of a solubility equilibrium between the precipitated and the dissolved iron species. The precipitated phases present in our system (Supplementary Table S1), which are also ubiquitous in aerobic, pH neutral environments (Kraemer, 2004), buffer the dissolved inorganic iron concentration to the solubility level. All dissolved iron sequestered by pyochelin will readily be replenished by dissolution of precipitated iron species. In contrast to this equilibrium scenario, we did observe growth inhibition in our experiments and conclude that pyochelin *can* inhibit a competitor because kinetically controlled processes govern the concentration of iron in solution, i.e., such dynamic systems are *not* in equilibrium with the precipitated iron (**Figure 2B**). Most importantly, the observed inhibition can be achieved if pyochelin binds soluble iron more rapidly than it is dissolving from the precipitated phases. In the following, we make a quantitative estimate of whether pyochelin could have sequestered significant amounts of dissolved iron to inflict growth inhibition. If, in the timeframe of the experiment, essentially none of the iron sequestered by pyochelin is replenished from precipitated iron, the 6 μM pyochelin we added can bind 3.1 nM of the dissolved iron under our experimental conditions (**Figure 2B**), and thus sequester around 40% of the total dissolved iron (7.9 nM, Supplementary Table S1). Consequently, the concentration of total dissolved inorganic iron (freely available without siderophore receptor) can theoretically decrease to a concentration of 5.9 nM. This reduction in dissolved inorganic iron would still allow the non-recipient to grow to around 7^∗^10^7^ cells per milliliter if each cell requires 5^∗^10^4^ iron atoms (the median of a range of published values for iron content per cell; Granger and Price, 1999; Andrews et al., 2003; Rolfe et al., 2012; Cunrath et al., 2015). However, assuming that iron uptake rates of the non-recipient depend on the concentration of dissolved inorganic iron (Campbell, 1996), this decrease in dissolved inorganic iron concentration would decelerate its growth enough to explain the growth dynamics observed (**Figure 1**).

Our model assumes that the non-recipient, which is incapable of siderophore production and uptake, only accesses dissolved, not precipitated iron. Note that the concentration of precipitated iron is almost 200-fold higher than the concentration of iron pyochelin could sequester in equilibrium (Supplementary Table S1). The added pyochelin would not be able to deplete this total iron pool sufficiently to explain the growth inhibition we observe. Therefore, we conclude that siderophore-independent iron uptake from precipitated iron sources, although reported before in *Pseudomonas* species (Kuhn et al., 2012), is unlikely on the relevant timescales in our system.

Overall, our experiments suggest that siderophores can induce growth inhibition efficiently in the presence of precipitated iron if the system is controlled by dissolution kinetics, even if the siderophore has a low iron-binding affinity and is present at low concentrations. Such kinetically controlled, saturated systems are potentially widespread in the environment, e.g., in marine high-nutrient-low-chlorophyll ocean areas where the iron concentration is controlled by mobilization rates from atmospheric dust inputs (Kraemer et al., 2005). Also, in saturated soil systems, dissolution kinetics have been described as a factor influencing the concentration of available iron (Schwertmann, 1991). Our hypothesis of kinetically mediated inhibition is consistent with the observation that the non-recipient started growing after a lag phase of several hours upon addition of pyochelin (**Figure 1A**). At longer incubation times, sufficient iron can be released from precipitated phases to support growth or, alternatively, the non-recipient can adapt to the low iron concentration (e.g., by increasing the number of transporters).

Dissolution kinetics can also explain the reduced inhibitory effect of pyochelin at a higher overall iron concentration in the system (30 μM FeCl_3_, **Figure 1A** and Supplementary Figure S1). Note, that the dissolved inorganic iron concentration remained the same, i.e., saturated, as in the system where less iron was added (1 μM FeCl_3_, Supplementary Table S1). The weaker inhibitory response was probably rather a consequence of increased amounts of precipitation that led to an increased surface area and thus faster iron oxide dissolution rates (Kraemer, 2004), given that the specific surface area is constant (Dzombak and Morel, 1990). Overall, our data show that siderophores can sufficiently achieve inhibition of a competitor in the presence of precipitated iron, however, only when the dissolution of precipitated iron is sufficiently slow.

### The Benefits of Pyochelin Secretion Are Primarily Based on Decreasing Iron Availability for Competitors

Based on the strong growth inhibition of the non-recipient observed in our first experiment, we were interested in how the two beneficial effects of siderophores, solubilization of precipitated iron and mediation of competition, compare to each other in our experimental system. Solubilization of precipitated iron species has been described as a key function of siderophores (Andrews et al., 2003). These two effects are not necessarily achieved by the same mechanism: If siderophores primarily bind iron from the precipitated phase, chelation of dissolved, freely available iron would be reduced, and hence also the competitive effect would be diminished. Here, we separately assessed the two distinct benefits of pyochelin: (a) increasing iron availability for growth and (b) decreasing iron availability for competitors.

**FIGURE 3:**
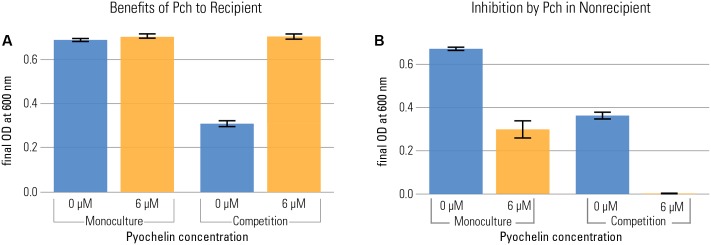
Pyochelin can be highly beneficial in competition, even when it is not beneficial in monoculture. Recipient **(A)** and non-recipient **(B)** were grown under iron limitation (1 μM FeCl_3_) either in monoculture or in 1:1 competition. The effect of pyochelin was tested by addition of 6 μM pyochelin standard (orange; control without pyochelin addition in blue). The benefit of pyochelin is quantified as the increase in yield of the recipient upon addition of pyochelin (i.e. increase from blue to yellow bar in plot a). The final yield of the recipient did not significantly increase upon addition of pyochelin in monoculture, but strongly increased in competition (two-way ANOVA shows a significant two-way interaction between pyochelin addition and social environment for the response variable final O.D., *F* = 442, *p* < 0.001). Likewise, in both culture conditions, the yield of the non-recipient without functional pyochelin receptor is reduced upon addition of pyochelin, but the effect is stronger in a competition. Barplots show the final yield of each strain reached after 20 h of growth, as O.D. at 600 nm (nine replicates per type; error bars depict the standard error of the mean). We calculated the final O.D. reached by each strain in competition by multiplying the final O.D. of the whole population (recipient + non-recipient) with the frequency of each strain as determined by flow cytometry.

To disentangle costs and benefits, we measured benefits of pyochelin in the recipient, a strain that does not produce pyochelin (but can take it up). We grew the recipient in the presence and absence of pyochelin in both monoculture and in competition with the non-recipient. We quantified the frequency of the strains in competition by flow cytometry, using strains that constitutively expressed fluorescent markers (P_A1/04/03_::*egfp* or *mcherry)*. To assess the benefit of pyochelin, we calculated the final optical density (O.D.) reached by each strain in competition and compared it to the final O.D. reached in monoculture. We found that, based on measuring yield after 20 h, the addition of pyochelin was only beneficial in competition: there was no increase in the recipient’s yield in monoculture, but a strong increase in competition (**Figure 3**). Of note, no significant increase in the growth rate of the recipient was observed upon addition of pyochelin (median growth rate of recipient without addition of pyochelin = 1.35 divisions per hour ± 0.029 standard error; median growth rate with 6 μM pyochelin of 1.42 divisions per hour ± 0.077 standard error; Kruskal–Wallis Rank Sum Test, chi-squared = 2, *p* = 0.2, **Figure 1A** and Supplementary Figure S1). Overall, our data support the hypothesis that the beneficial effect of pyochelin in this system is not linked to an increase in iron availability from precipitated sources, but rather to an inhibitory effect on the competing strain. We want to point out that the approach that we used to count the number of bacteria for the competition experiment – flow cytometry based on a constitutively expressed protein – does not allow to determine whether all the fluorescent cells are viable. It is possible some cells might still contain fluorescent protein although they are not viable anymore. As long as the fraction of dead cells is the same for different strains, this effect will only affect the absolute number of cells, but not their relative frequency as analyzed in **Figure 3**.

Note that the pyochelin operon has not been removed completely from the recipient strain (only the two pyochelin synthetases *pchE* and *pchF* have been deleted). Therefore, addition of pyochelin might induce production of pyochelin precursor molecules in the recipient strain that carries the functional receptor (Michel et al., 2007). However, we only compare benefits between monoculture and competition, where the recipient is exposed to the same level of pyochelin, hence any difference in growth between these two conditions is unlikely to be due to differences in costs of pyochelin-induced gene expression. Similarly, there might still be some transcription of genes coding for pyoverdine synthesis, since we used a strain only lacking one of the pyoverdine production genes, *pvdD*. This background is uniform for all of the strains used, though, so any difference between the strains should be independent of such effects.

In the competition study, we also found that the suppressive effect of pyochelin on the non-recipient’s growth was aggravated by the presence of the recipient. In monoculture, pyochelin reduced the yield of the non-recipient only by about a factor of two (addition of pyochelin reduced mean O.D. of non-recipient from 0.67 to 0.29). However, in competition and in the presence of pyochelin, the yield of the non-recipient dropped below the level of detection. Thus, a synergistic effect of pyochelin and a growing recipient strain on the non-recipient was apparent. Again, this enhanced effect can be explained by a kinetically mediated inhibition effect, given the assumptions we introduced before. We assume that recipient recycles pyochelin, as is the case for numerous siderophores (Ratledge and Dover, 2000). In the case of recycling, recipient cells take up the pyochelin-iron complex, remove the iron, and afterward release the unbound pyochelin again. Consequently, growing recipients constantly create unchelated pyochelin siderophores that bind freshly dissolved iron and significantly deplete the iron fraction in solution.

### Mediating Competition Could Be an Important Function of Pyochelin in the Presence of Precipitated Iron

**FIGURE 4:**
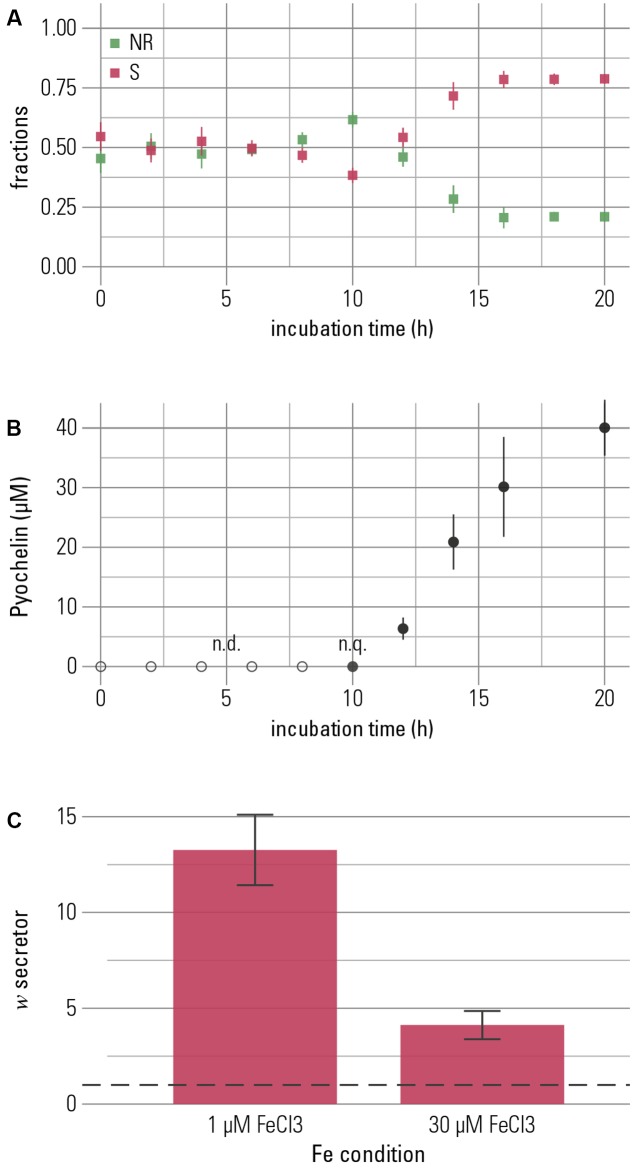
The competitive advantage of the pyochelin-secretor is temporally linked to production of pyochelin. Secretor and non-recipient were grown in 1:1-competitions for 20 h. **(A)** The fractions of each competition partner are shown over time with the secretor (S) in red and the non-recipient (NR) in green. The frequency of the secretor increased after 12 h of growth. **(B)** Increase of the secretor was temporally associated with the production of pyochelin. Pyochelin was detectable in the supernatant after 10 h and accumulated in the medium over time. Means of three biological replicates are shown and error bars represent standard error of the mean (n.q. = non-quantifiable, below quantification limit of 1 μM). **(C)** To quantify the overall outcome of competition, a proxy for relative fitness (*w*, Ross Gillespie et al., 2007), was calculated. Neutral competition outcome, where *w* equals 1, is indicated by a dashed line, and *w* > 1 indicates higher fitness of the secretor. The secretor shows a higher fitness at both low and high iron concentrations (1 μM FeCl_3_ and 30 μM FeCl_3_, respectively; Welch *t*-test with alternative hypothesis that true mean of *w* is greater than *μ* = 1: for 1 μM FeCl_3_ *t* = 6.67, *p* < 0.001, and for 30 μM FeCl_3_ *t* = 4.23, *p* = 0.002). Under iron-rich conditions, the increase of the secretor is smaller, though, than under iron-limitation (Wilcoxon rank sum test, *W* = 60.5, *p* = 0.003). This outcome is robust to the identity of the combination of strain and fluorescent protein (see Supplementary Figure S2). The bar chart shows the mean of eight repeats and error bars represent the standard error of the mean.

In the experiments described thus far, pyochelin was added to the medium. To investigate whether bacteria themselves would produce enough pyochelin to suppress competitors, we performed competition studies between a secretor and a non-recipient with an initial abundance ratio of 1:1. This experiment can yield further information on the functions of siderophores. If bacteria produce sufficient siderophores, iron sequestration could be an important function of pyochelin under these conditions, where we found growth promotion of a pyochelin-using strain to be mediated largely by competition, not iron solubilization (**Figure 3**). The two strains were distinguished on the basis of a constitutively expressed fluorescent protein as described above. At low total iron concentration (1 μM FeCl_3_), the relative frequency of non-recipient cells decreased after approximately 12 h, and this decrease was temporally associated with the onset of pyochelin production (**Figures 4A,B**). These observations support our hypothesis that growth inhibition is induced by pyochelin complexing available iron, i.e., pyochelin quickly binds dissolved iron, and this sequestration leads to a growth benefit for the secreting strain. Accordingly, the final relative frequency of the secretor increased (**Figure 4C**). A control competition experiment performed with the alternative fluorescent protein-strain combination led to the same qualitative outcome (Supplementary Figure S2).

In the presence of 1 μM ferric chloride, high amounts of pyochelin were produced (38.7 ± 4.6 μM, mean ± SEM, *n* = 3). In addition, we found that the secretor strain produced pyochelin also in the presence of high total iron concentrations (30 μM FeCl_3_). Although pyochelin production was significantly lower (Welch Two Sample *t*-test, *t* = 4.99, *p* = 0.01), considerable levels of secreted pyochelin were still maintained (10.5 ± 3.3 μM, mean ± SEM, *n* = 3). In addition, we detected an increase in the final relative frequency of the secretor strain (**Figure 4C**). We conclude that bacteria may be capable of producing enough siderophore to secure reservation of iron and engage in competitive inhibition under kinetically controlled conditions at both low and high total iron concentrations. Ultimately, siderophores might mediate competition for resources other than iron. If a competitor is inhibited in accessing iron, its reduced growth also translates into reduced consumption of other resources.

## Discussion

Our findings support the hypothesis that siderophores in environmental systems inhibit other bacteria that lack the specific siderophore receptor system, as supported by previous observational studies (Vachée et al., 1997; Gram et al., 1999; Khare and Tavazoie, 2015). We find that this inhibition is maintained in the presence of precipitated iron. Through thermodynamic modeling, we demonstrate that the ability of siderophores to efficiently deplete dissolved iron under these conditions depends on solution conditions. In the presence of precipitated iron, a condition that is typical for aerobic, pH-neutral environments, siderophores are able to inhibit competing strains only if kinetic processes govern the system. The following three conditions must be met to induce competitive inhibition in the presence of precipitated iron: (1) siderophores preferentially bind dissolved, not precipitated iron, (2) siderophores bind dissolved iron faster than iron can be replenished from the precipitated phase, and (3) iron uptake without siderophores present correlates with the concentration of dissolved, unchelated iron. We detected strong inhibitory effects even at low concentration of the weak siderophore pyochelin (pFe = 16, compared, e.g., to pyoverdine with pFe = 27; pFe being the negative logarithm of the concentration of ferric iron remaining unchelated in the presence of a standardized iron and siderophore concentration (Brandel et al., 2012). Considering this observed inhibition, we suggest that these three conditions mentioned above were met in the presented experimental system and in previously reported experimental settings where inhibition was reported (Khare and Tavazoie, 2015). While precipitation of insoluble iron is widespread, it is more challenging to quantify the equilibrium state of environmental systems, i.e., how often environmental systems are kinetically controlled. An example of environmental systems where siderophores were reported to achieve competitive inhibition are soil systems (Lemanceau et al., 2009), environments where kinetics of iron dissolution indeed seem to play a key role (Schwertmann, 1991).

In our experiments, the growth-promoting effect of pyochelin achieved through inhibition of a competing strain surpassed the effect of solubilizing precipitated iron. Possibly, these results would be different had we used a high-affinity siderophore that is more efficient at solubilizing the precipitated iron. However, our data show that, under conditions where solubilization of precipitated iron plays a minor role, a siderophore can have a large effect on competition. Hence, in some environments, mediating competition can be the dominant function of siderophore secretion.

Based on our findings, we suggest a new hypothesis regarding the presence of exogenous receptor genes in bacteria. Thus far, the evolutionary dynamics in siderophore production and uptake were mainly interpreted from the perspective of the public goods dilemma (Kümmerli and Brown, 2010; Cordero et al., 2012). For example, the observation that strains in the lungs of cystic fibrosis patients maintain siderophore receptors only as long as siderophore producers are present could indicate that strains with receptors are selected for because they cheat on producers and save siderophore production costs (Andersen et al., 2015). Our results suggest a complementary perspective, namely that the presence of siderophore producers forces other strains to maintain receptors to secure access to iron. Expression of a receptor makes siderophore-bound iron accessible, and renders bacteria immune against iron sequestration by competitors. We suggest that any strain losing the receptor gene will be strongly counterselected as long as siderophores are produced.

Overall, our findings underline the importance of siderophore secretion for antagonistic interactions, and show that the same biological trait may be important for cooperative as well as competitive interactions. Detecting genes encoding a given trait is thus not sufficient to draw firm conclusions about the relative importance of cooperative and competitive interactions in microbial communities. Rather, addressing this important question requires functional studies under conditions that are close to the natural settings.

## Author Contributions

KS, EJ, KM, and MA designed the research. SK developed the thermodynamic model. KS and EJ conducted the experiments. KS and MA conducted the statistical analyses. KS, EJ, SK, and MA wrote the manuscript.

## Conflict of Interest Statement

The authors declare that the research was conducted in the absence of any commercial or financial relationships that could be construed as a potential conflict of interest.
