# Supplementary material for: Magnitude and Mechanism of Siderophore-Mediated Competition at Low Iron Solubility in the Pseudomonas aeruginosa Pyochelin System
Source: Front Microbiol. 2017 Oct 10;8:1964. doi: 10.3389/fmicb.2017.01964 (PMC5649157; doi:10.3389/fmicb.2017.01964)
Supplement: Supplementary file 1 [file Data_Sheet_1.docx]

**Supplementary Information**

1. **Additional table**

| **Total [Fe]** | **dissolved inorganic [Fe]** | **pyochelin-bound [Fe]** | **precipitated [Fe]** |
| --- | --- | --- | --- |
| 1 µM | 7.9 nM | 5.2 nM | 0.99 µM |
| 30 µM | 7.9 nM | 5.2 nM | 29.99 µM |

**Table S1.** Concentration of iron species in equilibrium calculated by thermodynamic modeling. The pyochelin concentration is 6 µM in Succinate minimal medium at pH 7.2. The iron source is FeCl_3_.

1. **Additional data plots**


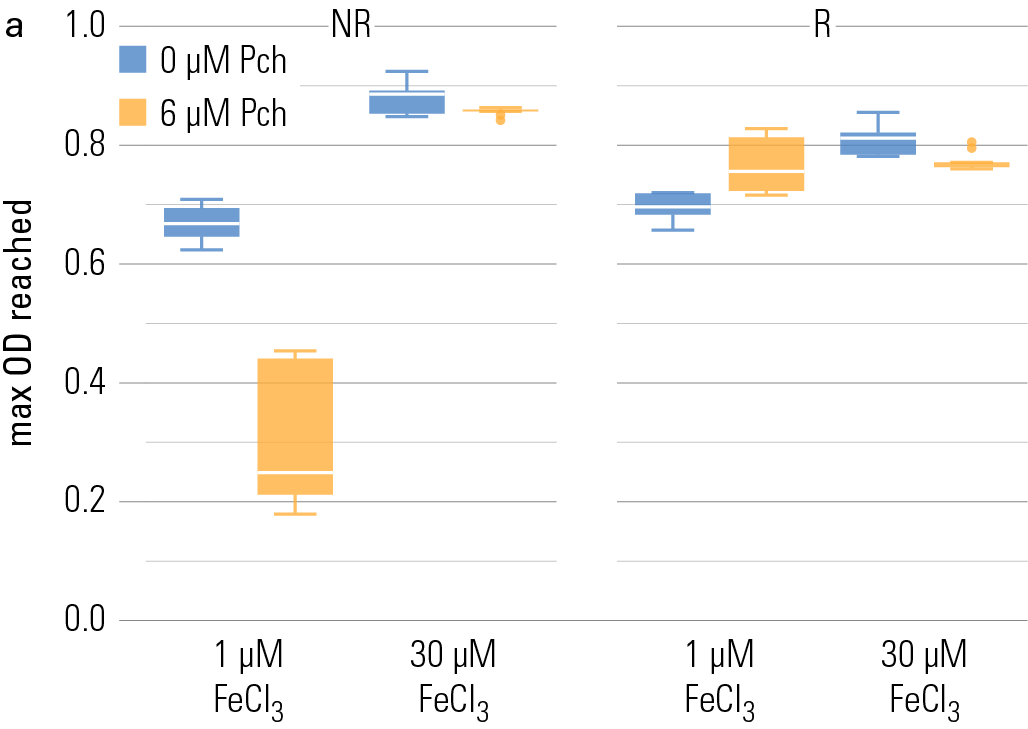


**
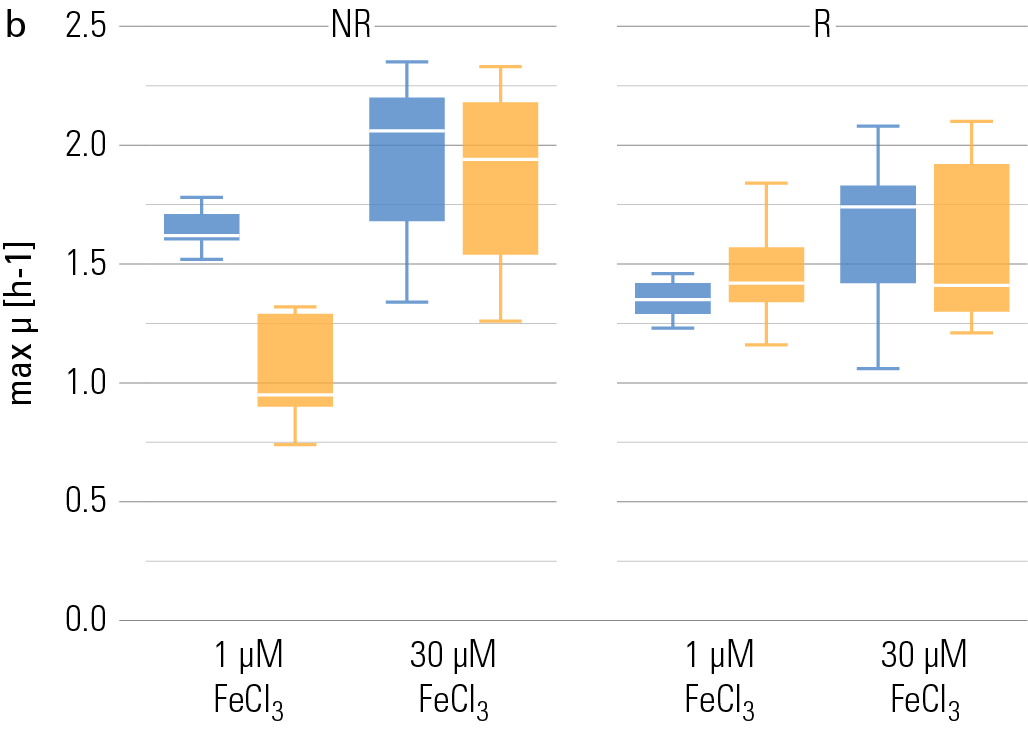
**

**
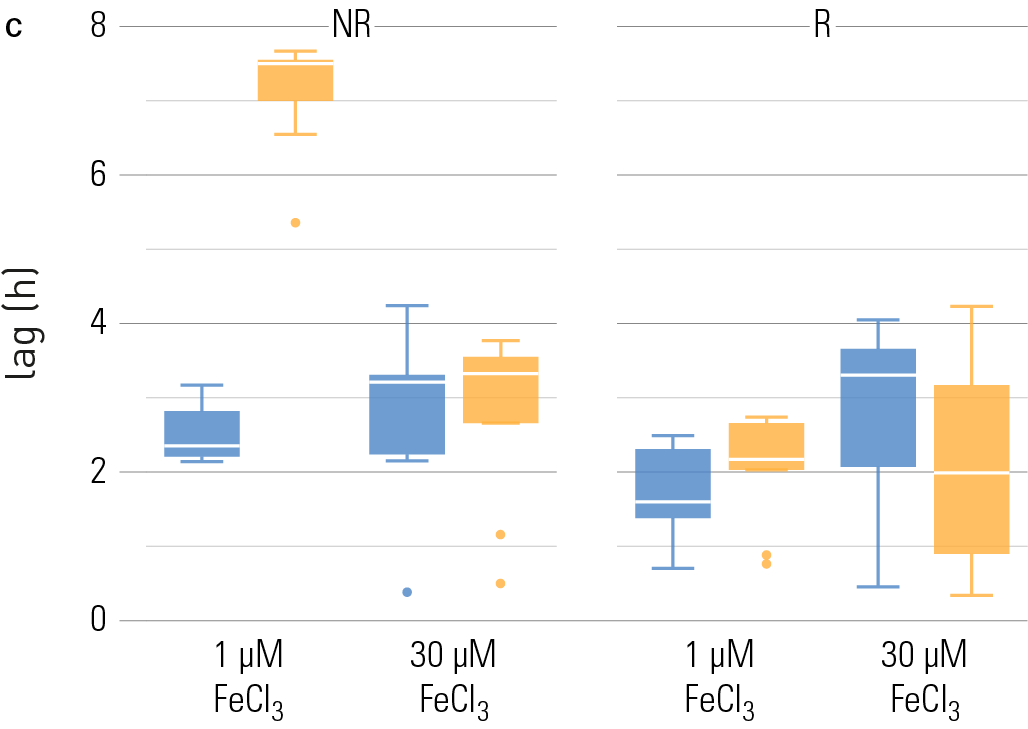
**

**Figure S1. Growth parameters of nonrecipient and recipient upon addition of pyochelin standard.** The plot illustrates the effect of pyochelin addition (6 µM for all data shown) on growth parameters of the nonrecipient (NR, left panel) and the recipient (R, right panel) (both shown in yellow) compared to the control without pyochelin addition (shown in blue). Data is shown for low and high iron concentrations (1 µM and 30 µM FeCl_3_, respectively) and for three growth parameters: a. effect on growth yield, i.e., maximal O.D. measured at 600 nm; b. effect on maximal growth rate; and c. effect on growth lag phase. The growth curves from which these growth parameters were estimated are shown in Fig. 1. Data in Figure S1 confirm the trend of growth curves presented in Figure 1: The nonrecipient is inhibited in all three growth parameters by addition of pyochelin in the iron-limited environment (3-way ANOVA shows a significant 3-way interaction between iron concentration, pyochelin addition, and strain, for all three response variables, for maximal growth rate F=9.1, p=0.004, for maximal yield F=122.1, p < 0.001, for lag F=17.3, p < 0.001).


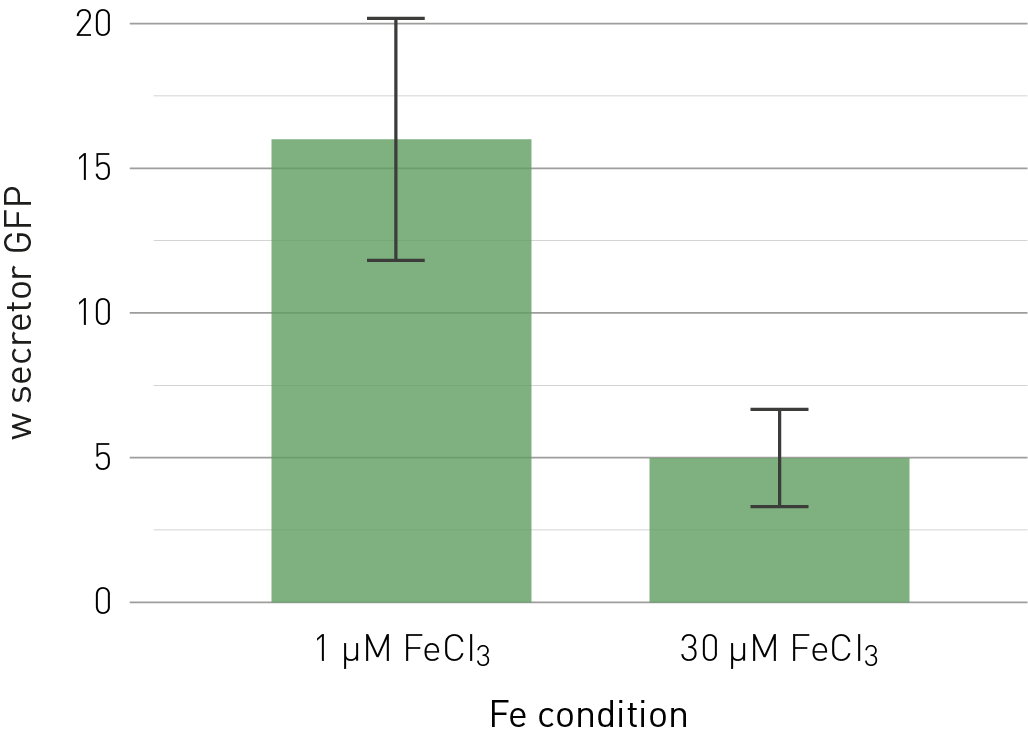


**Figure S2.** Relative fitness of a secretor tagged with *egfp* in competition with a nonrecipient tagged with *mcherry* after 20 hours of growth. As in the competition with the alternative fluorescent protein-strain combination (Fig. 4c), the secretor increases significantly in fitness *w* (Ross-Gillespie *et al.*, 2007) at low and high iron concentrations (Welch t-test with alternative hypothesis that true mean of *w* is greater than *µ*=1: for 1µM FeCl_3_ t=3.6, p=0.004, and for 30 µM FeCl_3_ t=2.4, p=0.03). The bar chart shows the mean of eight replicates and error bars represent the standard error of the measurements.

**(3) Additional method details**

**Quantification and statistical analysis of growth parameters in monoculture studies.** To assess the effect of pyochelin on the nonrecipient and the recipient in monoculture, we used O.D. measurements to deduct the growth parameters maximal growth rate and lag time. The background O.D. was determined from non-inoculated wells at the periphery of the plate. The maximal specific growth rate was estimated by an exponential fit of background-subtracted O.D. values within the O.D.-range of 0.01 to 0.1 (Equation 1, Matlab script written by Daan Kiviet, ETH Zurich)

OD(t) = OD_0_·2^(µ(max)·t)^ , (1)

where *µ(max)* is the exponential growth rate and *OD_0_* is the O.D. at the beginning of the experiment, i.e., the calculated inoculum concentration. The lag phase was estimated by extrapolating the tangent at the exponential part of the growth curve back to the inoculum level OD_inoc_ (Equation 2, as suggested in (Swinnen *et al.*, 2004)):

t_lag_ = 2·log(OD_inoc_) / *µ_max_* (2)

**Conduction of time course studies**

For this experiment (results shown in Figure 5), a larger growth volume was necessary to isolate pyochelin. Since we noticed that the competitions performed in glass Erlenmeyer flasks did not lead to an inhibitory effect, probably due to iron contamination by the glass surface, we set up a design where we distributed the initial medium in ten 50 ml-Falcon tubes and sacrificed one tube for each measurement. Also, since we followed growth over 20 hours, we set up two runs in parallel, one for the time points until 12 hours, and another one from 14 hours until 20 hours. Both runs were started from the same streaked clone.

**Statistical analyses.**

For monoculture growth estimates (results Figure 1, S6), we used a 3-way ANOVA in SPSS, with either maximal growth rate, maximal O.D. reached, or lag phase as response variable and strain identity (2 levels: recipient, nonrecipient), iron concentration (2 levels: 1 µM FeCl_3_, 30 µM FeCl_3_), and pyochelin (2 levels: 0 µM pyochelin, 6 µM pyochelin) as explanatory factors. The date of the experiment (3 levels) was included as a random factor. We tested a model including a 3-way interaction of all factors. For all response variables, data were not homoscedastic, but we were not able to find a transformation to overcome this limitation.

For analysis of the benefits obtained by addition of pyochelin (Figure 3), we used a 2-way ANOVA in SPSS. Final O.D. reached by the recipient strain was the response variable, and pyochelin (2 levels: 0 µM pyochelin, 6 µM pyochelin) and environment (2 levels: monoculture, competition) were the explanatory variables. The date of the experiment (3 levels) was included as a random factor. We tested a model including a 2-way interaction of the factors. The data were not homoscedastic, but we were not able to find a transformation to overcome this limitation.

For analysis of relative fitness (Figure 4), we used the Wilcoxon-Rank Sum in R (R Core Team (2014). R: A language and environment for statistical computing. R Foundation for Statistical Computing, Vienna, Austria. https://www.R-project.org/) to test whether iron concentration (2 levels: 1 µM , 30 µM) had a significant effect on relative fitness *w*. We did this for each combination of fluorescent proteins separately (recipient:GFP – secretor:mcherry, and vice versa). To assess whether relative fitness is significantly different from 1, the neutral competition outcome, we performed a Welch t-test with the alternative hypothesis that the true mean of w is greater than µ=1 for each fluorescent protein combination separately.

**(5) Additional methods: Measurement of pyochelin with HPLC**

**Development of pyochelin detection method**

We developed the detection of pyochelin with a commercially available standard (EMC microcollections, Tübingen, Germany) and reversed phase high-pressure liquid chromatography (rp-HPLC, 1200 series, Agilent Technology). In a first step, we established a detection method for pyochelin by absorption of the complex of pyochelin with iron (pch-Fe) based on previously published protocols (Adler *et al.*, 2012; Youard *et al.*, 2007). Measurements using free pyochelin can result in complications, since free pyochelin is present as two interconvertible diastereomers (Ankenbauer and Cox, 1988), and can be complexed by a variety of metals (Cox and Graham, 1979), factors which influence retention times and absorption peak height in the rp-HPLC analysis (Fig. S3a). However, only one of the diastereomers binds iron (Hayen and Volmer, 2006; Schlegel *et al.*, 2004) and in the presence of iron, pyochelin is driven towards this type of diastereomer (Youard *et al.*, 2007). Thus, we incubated all pyochelin samples prior to quantification with 10 mM FeCl_3_ for at least 15 hours at 4°C in the dark. After incubation with FeCl_3_ one peak with absorbance at 250 nm and 520 nm dominates in our HPLC analysis (Fig. S3) indicative of the pyochelin-iron complex (pch-Fe) (Cox and Graham, 1979).

For the detection of pch-Fe by rp-HPLC, 30 µL sample were injected onto a C8 column (Eclipse XDB-C8, 5 µm, 4.6 x 150 mm, Agilent) and a gradient method was used with (A) 0.1% trifluoroacetic acid (TFA) (Prolabo, 99% purity, pH 1.8) and (B) acetonitrile (Merck, HPLC-grade, containing 0.1% TFA) by increasing (B) from 6% to 60% within 10 minutes at room temperature and a flow rate of 1 mL min^-1^. The pch-Fe complex was identified by absorbance as the peak with a retention time of 7.3 minutes (± 0.04 minutes). The area of this peak at 250 nm increased linearly with increasing concentration of pyochelin standard added (r^2^=1, p<0.001) (Fig. S4). This compound was not detected in control extractions of supernatant from a nonrecipient strain, which was genetically not able to produce pyochelin. Therefore, we identified this peak as the pch-Fe complex and used it to quantify pyochelin for all presented experiments.


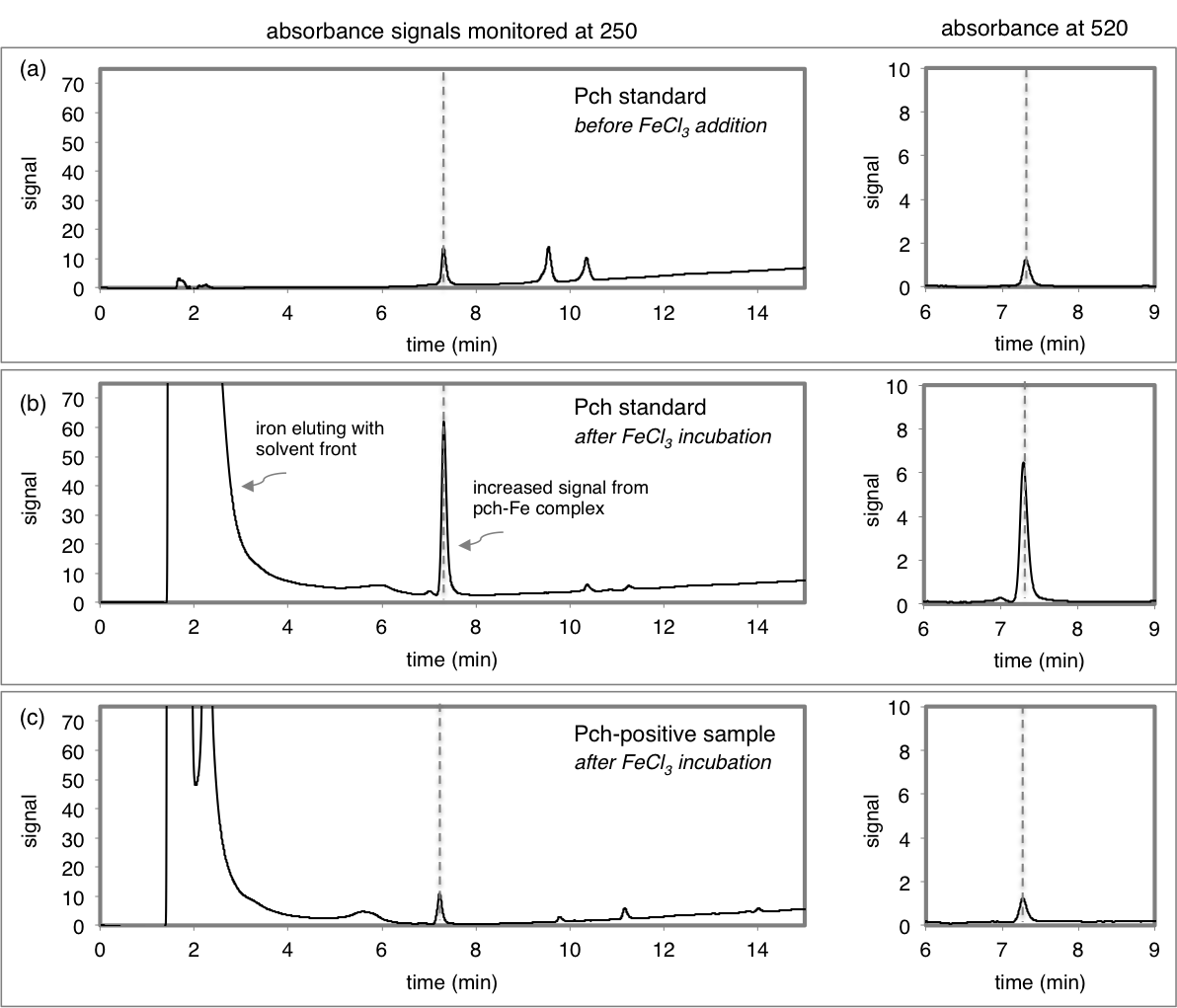


**Figure S3.** Effect of iron addition on pyochelin detection. HPLC chromatogram of (a) pyochelin (Pch) standards before, and (b) after incubation with iron chloride (10 mM FeCl_3_), (c) extract of secretor culture capable of producing Pch after incubation with FeCl_3_. The Pch-Fe complex is detected at 7.3 min retention time and exhibits absorbance at 250 nm and 520 nm (chromatograms to the left and right, respectively). When measuring at 520 nm, no other significant peaks were measurable; therefore a zoom on the one peak is shown.


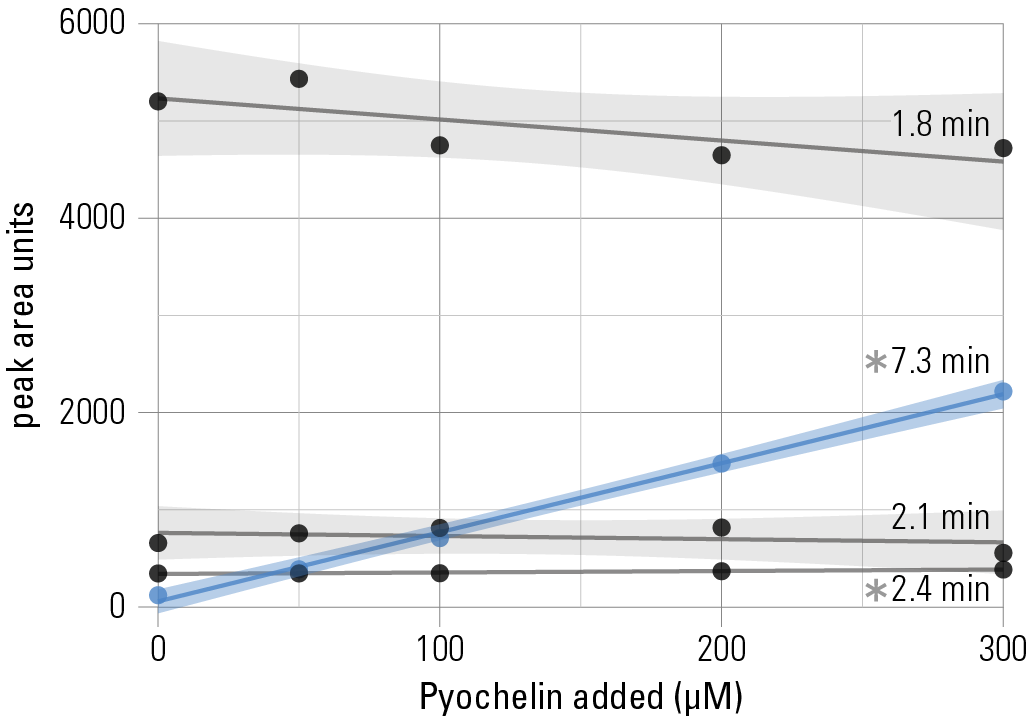


**Figure S4.** Identification of the pyochelin-iron complex in rp-HPLC. Standard addition with pyochelin standard was performed to the diluted extract from an overnight secretor culture, i.e., in a representative matrix, and incubated with 10 mM FeCl_3_. All areas of those peaks are shown detected at 250 nm (retention times are noted accordingly). Each data point represents one measurement and the shaded area indicates the standard error of a linear regression fit. Two peaks increased significantly upon addition of pyochelin standard, indicated by an asterisk, with retention times of 2.4 min (r^2^=0.96, p=0.002) and of 7.3 min (r^2^=0.99, p<0.001). We only used the peak at 7.3 min, indicated in blue, for our analyses, since the 2.4 min peak is part of the iron effluent eluting with the solvent front (compare data in Fig. S3 b and c).

**Pyochelin standard addition and limit of quantification**

To quantify the concentration of pyochelin in the extracts of supernatant of bacterial cultures, we performed a standard addition. Four to six steps of increasing concentrations of pyochelin standard were added to the extracts and analyzed by rp-HPLC. The standard additions ranged between two to ten times the pyochelin concentrations present in the sample. The x-intercept, corresponding to the pyochelin concentration originally present in the extract, was calculated using linear regression on the peak areas over added pyochelin concentrations (Fig. S5).

The limit of quantification is set by (a) the ability to detect and integrate peaks in the HPLC and (b) the signal strength compared to the error introduced by the standard addition calculations. We estimated the limit of quantification by using the lowest peak we measured overall, from the time course measurements time point 10 h, and calculated the standard error of this standard addition curve, using the algebraic method (Bruce and Gill, 1999). We were able to detect a pyochelin concentration as low as 0.33 ± 0.137 μM SEM. Our approximate limit of quantification was thus 0.33 μM plus 5 times the SEM, i.e., 0.33 ± 0.683 μM; yielding a quantification limit of around 1 μM pyochelin.


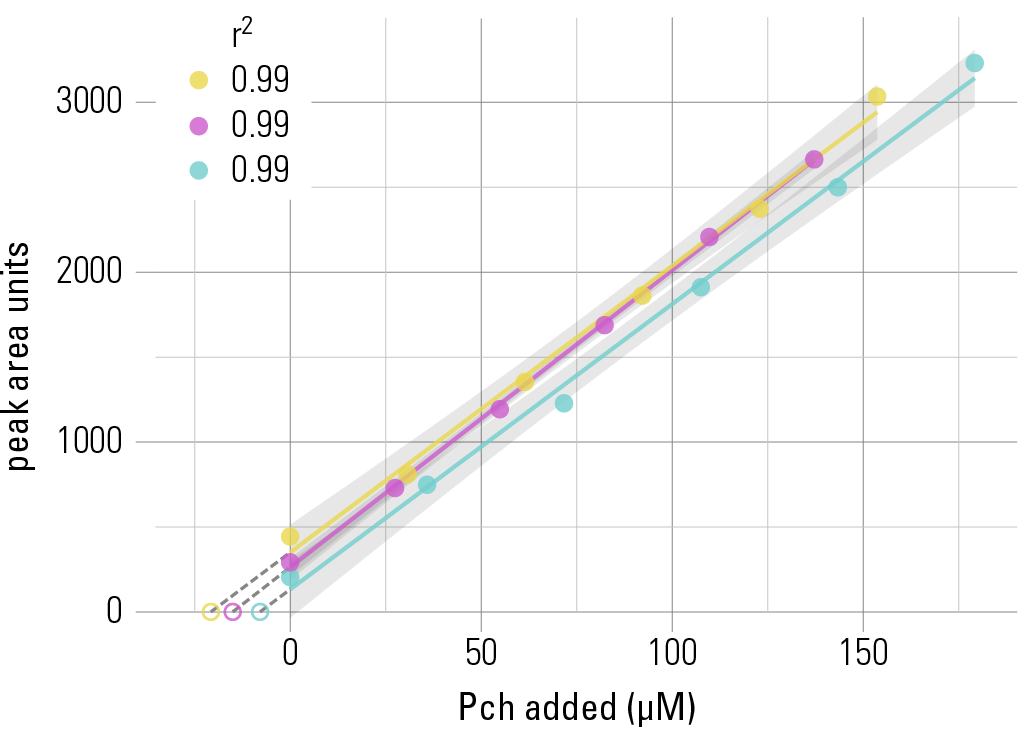


**Figure S5.** Example of standard addition method used for determining pyochelin (Pch) concentration. Extracts were made from secretor and nonrecipient competition cultures grown in succinate minimal medium with 1 μM FeCl_3_ for 20 hours and were incubated with 10 mM FeCl_3_. Three biological replicates of pyochelin extracts are shown and lines represent a linear regression fit with the standard error included as grey area (the coefficients of determination are shown in the top left). The pyochelin concentration originally in the sample was deducted as the x-intercept of the predicted linear fit, shown as open circles.

**Determining concentration of the pyochelin standard**

Pyochelin standard solutions were obtained from EMC microcollections (Tübingen, Germany). For the HPLC analysis, two different pyochelin stocks were used. We determined the concentration of each pyochelin stock solution using the extinction coefficient from (Braud *et al.*, 2009) measured for the absorbance at 310 nm following the Beer-Lambert Law (Fig. S6). The concentrations of the supplied pyochelin stock solutions varied by approximately a factor of four. We used the concentrations determined based on molar absorptivity for the standard addition calculations.


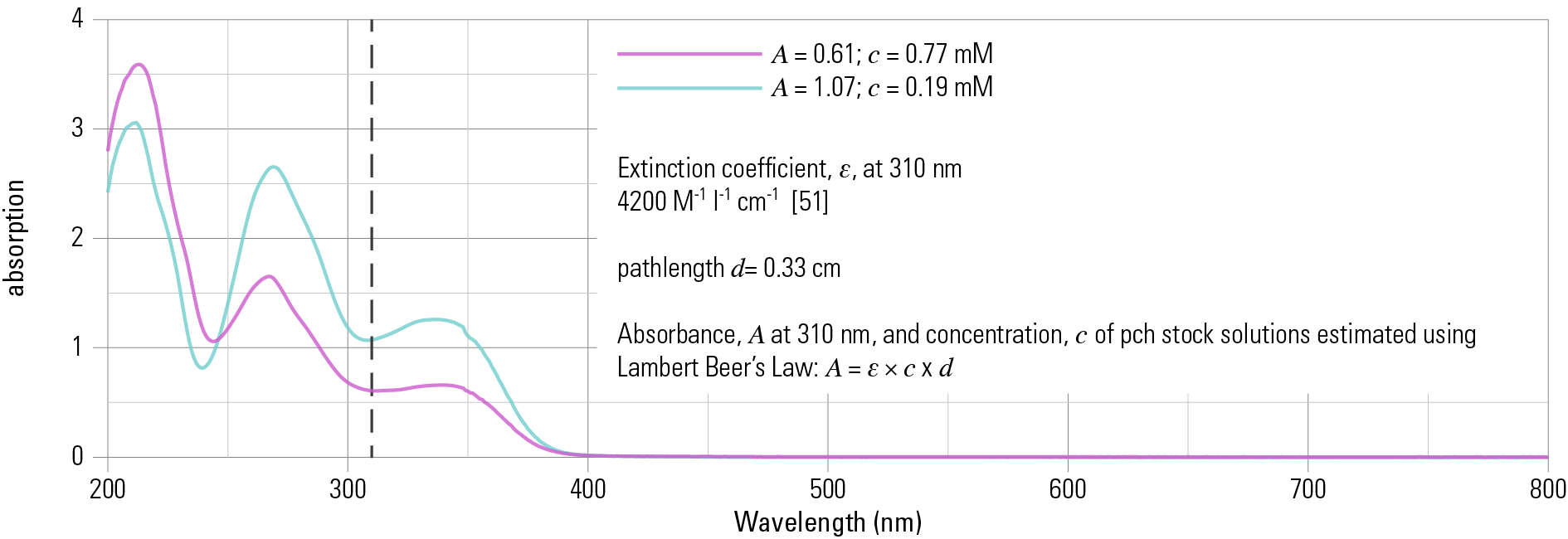


**Figure S6.** The absorption spectra of the two pyochelin standard stock solutions used for our experiments, measured as dilutions in HPLC-grade methanol. The absorbance at 310 nm, indicated by the dashed line, was used for calculation of pyochelin concentration. Details on the calculation and calculated concentration of each stock are shown as inset.

**Sensitivity limits of pyochelin extraction**

For all pyochelin measurements, three extraction rounds were performed. To evaluate how quantitative the pyochelin extraction from supernatants was, we measured pyochelin concentrations in a total of four consecutive extraction rounds and fitted an accumulation curve (Fig. S7). To obtain higher total pyochelin concentrations for these tests, extracts of those cultures were used that were grown under stronger iron-limiting conditions than used for the other experiments, i.e., no iron was added to the medium. This evaluation represents a conservative extraction estimate, since the culture conditions used for the other experiments contained lower total amounts of pyochelin. From the data presented in Figure S7, we estimate that with the three extraction rounds, we captured a substantial fraction, around 93.6%, of the total pyochelin present.


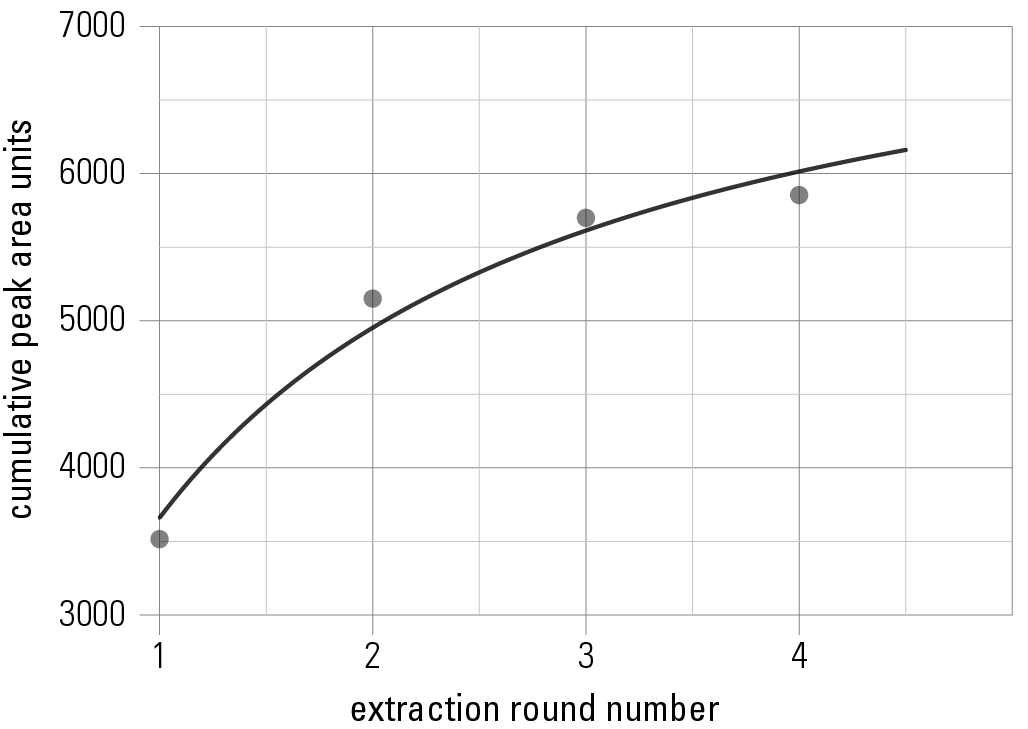


**Figure S7.** The cumulative area of the pyochelin-specific peak is plotted against the extraction round. Based on richness estimator methods used for estimating species richness in ecology studies (Hughes *et al.*, 2001) as well as extraction efficiencies in biochemical studies (Janos *et al.*, 2008), we fitted accumulation curves to extrapolate the total amount of pyochelin present in the sample. We fitted both a Michaelis-Menten function as the accumulation curve as well as an asymptotic regression with the lower limit of 0, using the drc package in R (Ritz and Streibig, 2005). The asymptotic regression gave a significantly better fit (p<0.001, LogLik =-27.54 versus LogLik=-31.67, shown as fitted line) and from this curve we estimated the total cumulative area of pyochelin theoretically present in the extract (with an area of 6093 +/- 83.6).

**References**

Adler C, Corbalán NS, Seyedsayamdost MR, Pomares MF, de Cristóbal RE, Clardy J, *et al.* (2012). Catecholate Siderophores Protect Bacteria from Pyochelin Toxicity Liles, MR (ed). *PLoS One* **7**: e46754.

Andrews SC, Robinson AK, Rodríguez-Quiñones F. (2003). Bacterial iron homeostasis. *FEMS Microbiol Rev* 215–237.

Ankenbauer RG, Cox CD. (1988). Isolation and characterization of Pseudomonas aeruginosa mutants requiring salicylic acid for pyochelin biosynthesis. *J Bacteriol* **170**: 5364–5367.

Braud A, Hannauer M, Mislin GLA, Schalk IJ. (2009). The Pseudomonas aeruginosa Pyochelin-Iron Uptake Pathway and Its Metal Specificity. *J Bacteriol* **191**: 3517–3525.

Bruce GR, Gill PS. (1999). Estimates of precision in a standard additions analysis. *Journal of Chemical Education* **76**: 805.

Campbell PGC. (1996). Interactions Between Trace Metals and Aquatic Organisms: A Critique of the Free Ion Activity Model. In: *Metal Speciation and Bioavailability in Aquatic Systems*, Tessier, A & Turner, RD (eds) Vol. 3, John Wiley & Sons: New York, pp 45–102.

Cox CD, Graham R. (1979). Isolation of an iron-binding compound from Pseudomonas aeruginosa. *J Bacteriol* **137**: 357–364.

Cunrath O, Geoffroy VA, Schalk IJ. (2015). Metallome of Pseudomonas aeruginosa: a role for siderophores. *Environ Microbiol* doi: 10.1111–1462–2920.12971.

Granger J, Price N. (1999). The importance of siderophores in iron nutrition of heterotrophic marine bacteria. *Limnol Oceanogr* **44**: 541–555.

Hayen H, Volmer DA. (2006). Different iron-chelating properties of pyochelin diastereoisomers revealed by LC/MS. *Anal Bioanal Chem* **385**: 606–611.

Hughes JB, Hellmann JJ, Ricketts TH, Bohannan BJM. (2001). Counting the Uncountable: Statistical Approaches to Estimating Microbial Diversity. *Appl Environ Microbiol* **67**: 4399–4406.

Janos DP, Garamszegi S, Beltran B. (2008). Glomalin extraction and measurement. *Soil Biol Biochem* **40**: 728–739.

Kraemer SM. (2004). Iron oxide dissolution and solubility in the presence of siderophores. *Aquatic Sciences - Research Across Boundaries* **66**: 3–18.

Malone JG, Jaeger T, Spangler C, Ritz D, Spang A, Arrieumerlou C, *et al.* (2010). YfiBNR Mediates Cyclic di-GMP Dependent Small Colony Variant Formation and Persistence in Pseudomonas aeruginosa Roy, CR (ed). *PLoS Pathog* **6**: e1000804.

Ritz C, Streibig JC. (2005). Bioassay analysis using R. *J STAT SOFTW*.

Rolfe MD, Rice CJ, Lucchini S, Pin C, Thompson A, Cameron ADS, *et al.* (2012). Lag Phase Is a Distinct Growth Phase That Prepares Bacteria for Exponential Growth and Involves Transient Metal Accumulation. *J Bacteriol* **194**: 686–701.

Ross-Gillespie A, Gardner A, West SA, Griffin AS. (2007). Frequency Dependence and Cooperation: Theory and a Test with Bacteria. *Am Nat* **170**: 331–342.

Schlegel K, Taraz K, Budzikiewicz H. (2004). The stereoisomers of pyochelin, a siderophore of Pseudomonas aeruginosa. *Biometals* **17**: 409–414.

Swinnen IAM, Bernaerts K, Dens EJJ, H GA, Van Impe J F. (2004). Predictive modelling of the microbial lag phase: a review. *Int J Food Microbiol* **94**: 137–159.

Youard ZA, Mislin GLA, Majcherczyk PA, Schalk IJ, Reimmann C. (2007). Pseudomonas fluorescens CHA0 Produces Enantio-pyochelin, the Optical Antipode of the Pseudomonas aeruginosa Siderophore Pyochelin. *J Biol Chem* **282**: 35546–35553.
